# Predicting herbivore faecal nitrogen using a multispecies near-infrared reflectance spectroscopy calibration

**DOI:** 10.1371/journal.pone.0176635

**Published:** 2017-04-28

**Authors:** Miriam Villamuelas, Emmanuel Serrano, Johan Espunyes, Néstor Fernández, Jorge R. López-Olvera, Mathieu Garel, João Santos, María Ángeles Parra-Aguado, Maurizio Ramanzin, Xavier Fernández-Aguilar, Andreu Colom-Cadena, Ignasi Marco, Santiago Lavín, Jordi Bartolomé, Elena Albanell

**Affiliations:** 1 Servei d’Ecopatologia de Fauna Salvatge, Departament de Medicina i Cirurgia Animals, Facultat de Veterinária, Universitat Autònoma de Barcelona (UAB), Bellaterra, Barcelona, Spain; 2 Department of Agronomy, Food, Natural Resources, Animals and Environment (DAFNAE), University of Padova (UNIPD), Agripolis, Legnaro, Italy; 3 Departamento de Biologia & CESAM, Universidade de Aveiro (UA), Aveiro, Portugal; 4 Department of Conservation Biology, Estación Biológica de Doñana, Consejo Superior de Investigaciones Científicas EBD-CSIC, Sevilla, Spain; 5 Office National de la Chasse et de la Faune Sauvage (ONCFS - Unité Faune de Montagne), Gières, France; 6 Sanidad y Biotecnología (SaBio), Instituto de Investigación en Recursos Cinegéticos, IREC (CSIC-UCLM-JCCM), Ciudad Real, Spain; 7 Ruminant Research Group, Departament de Ciència Animal i dels Aliments, Universitat Autònoma de Barcelona (UAB), Bellaterra, Barcelona, Spain; Tokai University, JAPAN

## Abstract

Optimal management of free-ranging herbivores requires the accurate assessment of an animal’s nutritional status. For this purpose ‘near-infrared reflectance spectroscopy’ (NIRS) is very useful, especially when nutritional assessment is done through faecal indicators such as faecal nitrogen (FN). In order to perform an NIRS calibration, the default protocol recommends starting by generating an initial equation based on at least 50–75 samples from the given species. Although this protocol optimises prediction accuracy, it limits the use of NIRS with rare or endangered species where sample sizes are often small. To overcome this limitation we tested a single NIRS equation (i.e., multispecies calibration) to predict FN in herbivores. Firstly, we used five herbivore species with highly contrasting digestive physiologies to build monospecies and multispecies calibrations, namely horse, sheep, Pyrenean chamois, red deer and European rabbit. Secondly, the equation accuracy was evaluated by two procedures using: (1) an external validation with samples from the same species, which were not used in the calibration process; and (2) samples from different ungulate species, specifically Alpine ibex, domestic goat, European mouflon, roe deer and cattle. The multispecies equation was highly accurate in terms of the coefficient of determination for calibration R^2^ = 0.98, standard error of validation SECV = 0.10, standard error of external validation SEP = 0.12, ratio of performance to deviation RPD = 5.3, and range error of prediction RER = 28.4. The accuracy of the multispecies equation to predict other herbivore species was also satisfactory (R^2^ > 0.86, SEP < 0.27, RPD > 2.6, and RER > 8.1). Lastly, the agreement between multi- and monospecies calibrations was also confirmed by the Bland-Altman method. In conclusion, our single multispecies equation can be used as a reliable, cost-effective, easy and powerful analytical method to assess FN in a wide range of herbivore species.

## Introduction

Faeces from mammal species are relatively abundant and easily collected. They provide valuable biological information on both existing [1,2] and extinct species [3,4]. On the other hand, faecal pellets can remain on the ground for months [5,6] and relevant biological information such as DNA markers [7] or dietary attributes [8] can be accurately assessed up to one week post-defecation.

Near-infrared reflectance spectroscopy (NIRS) has recently become very popular among foraging ecologists interested in analysing faecal contents [9–11]. Among other advantages, NIRS offers quick, non-destructive and reliable quantitative analyses of a broad range of organic constituents [12], including indicators of the diet quality of domestic and wild animal species [13,14]. As a result, diet quality assessment of free-ranging ungulate populations is one of the typical questions addressed by faecal analysis [15].

Among indicators to assess diet quality, faecal nitrogen (FN) is the most common by far. The protein content of plants and forage digestibility are directly related [16,17], as shown by the high correlation between the chemical composition of forages and FN in animals feeding at the same place [9,18,19]. On the other hand, there is experimental evidence of a relationship between the nitrogen provided by the food and the residual nitrogen excreted in faeces in ruminants [20]. This makes FN a good proxy for diet quality linked to overall diet digestibility [21], which has been used for more than 20 years as a nutritional index for mammalian herbivores [22–27], and in particular for ungulates [28].

However, efficiency in food digestion, or digestibility depends on both the quality of the food and the ecophysiology of the consumer [29]. In fact, an increase in gut capacity allows animals to process more food of lower digestibility in order to meet their energetic requirements, which in turn increases with their body weight. As a consequence, large herbivores are able to process less digestible food than their medium or small size counterparts [21]. For this reason, ungulate species have contrasting digestive physiologies and diet selections [30–32] that, in turn, may have seasonally changing faecal components [2,33]. For all of the previously mentioned reasons, researchers have assumed that the assessment of the diet through faeces must be species-specific, and thus faecal NIRS equations for one species may not be applicable to another [18].

In practical terms, a minimum of 50 to 75 independent samples should be considered for NIRS calibrations from small and homogeneous populations. However, if the equation is expected to be used on a large and diverse population, then a minimum of 150 samples is required for calibration [34,35]. As a rule of thumb, it is commonly accepted that a larger number of calibration samples generally lead to increased precision in NIRS predictions [36]. This fact imposes a large constraint on the use of NIRS for assessing diet quality on rare, elusive or declining mammal populations, where sample size is likely to be low. In other domains, however, this limitation has been solved with the elegant use of multispecies calibrations. In other words, a single equation made with samples from different species can be used to predict in a broad range of situations. These multispecies calibrations have been performed to assess chemical composition of forages [37] and grasses [38] or degradation parameters of feedstuffs [39,40]. To the best of our knowledge the approach using calibrations composed from multiple species has not been used to predict faecal composition in mammalian herbivores.

In this research, we developed an NIRS multispecies equation to predict diet quality, as assessed by the FN of five herbivores with highly contrasting digestive physiologies: two hindgut fermenters, (European rabbit *Oryctolagus cuniculus* and horse *Equus caballus*), and three foregut fermenters (Pyrenean chamois *Rupicapra pyrenaica*, red deer *Cervus elaphus*, and sheep *Ovis aries*). We first carried out individual FN calibration equations for each species (monospecies calibration equations) and then performed a global calibration equation including all species (multispecies calibration equation). Subsequently, we tested if this FN multispecies calibration equation could be applied to predict the FN content from other species of herbivores (e.g. Alpine ibex *Capra ibex*, cattle *Bos taurus*, domestic goat *Capra hircus*, European mouflon *Ovis musimon*, and roe deer *Capreolus capreolus*), which were not included in the original multispecies calibration. We propose that the FN amounts of the excluded species (validation species) are within the range of the species used to build our multispecies equation (calibration species) and hence, the FN from these new samples should be predicted with accuracy.

## Materials and methods

### Ethics statement

Since this study did not involve endangered or protected species, no specific permissions were required. Faecal samples were collected from the ground or from hunter harvested individuals such as red deer, roe deer and mouflon. In the present study, these three ungulate species were legally hunted in their own habitat by authorized gamekeepers and hunters within the framework of scientific programs approved by the comptetent authorities (e.g. French Ministry of Environment for roe deer and mouflon) or annual hunting plans approved by France, Portugal and Spain.

### Sampling collection and procedure

The faecal samples were collected from different geographic areas. They were taken from nine different species of herbivores and as much as possible at different time of year. Faecal samples were from both males and females as well as from young and adult animals (Table 1). This sampling procedure was used to obtain a general overview of the biological variability occurring in the field and provided us with a highly versatile collection of feeding behaviours (grazers or browsers), energetic requirements in relation to feed intake (body size) and digestive physiologies (ruminants or post-gastric fermenters). We used faecal samples of five free-ranging herbivores, specifically the European rabbit, horse, Pyrenean chamois, red deer and sheep. Moreover, to test the performance of the multispecies calibration in other herbivore species with similar digestive physiology, samples from Alpine ibex, cattle, domestic goat, European mouflon and roe deer were also used.

**Table 1:** Species and sources of faecal samples used in NIRS calibration equations to predict faecal nitrogen.

Species	Country	Location
Horse (*Equus ferus)*	Spain	Freser-Setcases National Game Reserve, Eastern Pyrenees
Sheep (*Ovis aries)*		
Pyrenean chamois (*Rupicapra pyrenaica*)		
Red deer (*Cervus elaphus)*	Portugal	Lombada National Hunting Area, Cubeira Tourist Hunting Area, Herdade da Negrita Norte Tourist Hunting Area and Lousã Mountain
	Spain	Sierra de la Culebra Regional Hunting Reserve, Montes Universales Hunting Reserve, Doñana National Park, Caspe/Fraga Social Hunting Areas and Quintos de Mora Hunting Area
European rabbit (*Oryctolagus cuniculus)*	Spain	Estación Biológica de Doñana, CSIC, Sevilla
Alpine ibex (*Capra ibex)*	Italy	Marmolada massif, Eastern Italian Alps,by the Department of Agronomy, Food, Natural Resources, Animals and Environment. University of Padova, Padova
Goat (*Capra hircus)*	Spain	Farm of Faculty of Veterinary of the Universitat Autònoma de Barcelona
Cattle (*Bos taurus)*		
European Mouflon (*Ovis musimon)*	France	Caroux-Espinouse massif,Office National de la Chasse et de la Faune Sauvage
Roe deer (*Capreolus capreolus)*		

Fresh droppings were collected from latrines (European rabbit), from the ground after observing individuals defecating (horse, sheep, domestic goat, cattle, Alpine Ibex, Pyrenean chamois), directly from hunter-harvested animals (Pyrenean chamois, European mouflon, red deer) and from animals physically restrained for research purposes (roe deer). In all cases, the droppings were stored in individual plastic bags, transported with refrigeration at 4°C and stored frozen at −20°C until FN determination. The species and the sampling date were recorded. Fresh samples were chosen in the field according to their colour and texture [6]. In the hunted animals, faeces samples were extracted directly from the rectum. In the field, a group of droppings collected in one bag was considered a faecal sample, even if they belonged to different individuals.

In the laboratory, frozen faeces were thawed and oven-dried at 60°C to constant weight (24 hours) and subsequently ground with a laboratory mill to a pitch of 1mm (Cyclotec 1093, FOSS Tecator, Höganäs, Sweden). Subsamples were used in duplicate to determine the dry matter by drying at 103°C and the FN content. The percentage of FN on a dry matter basis (%FN/DM) was determined by the Dumas dry combustion method, using a LECO analyser (LECO Corporation, St. Joseph, MI, USA). Faecal samples were dried and analysed according to AOAC protocols [41].

### NIRS analysis and calibration procedure

Ground faecal samples were packed in ringcups sample cells and were then scanned from 1,100 to 2,500 nm using a NIRSystems 5000 scanning monochromator (FOSS, Hillerød, Denmark). Reflectance was recorded at 2 nm intervals as log (1/R), where R represents the reflected energy, resulting in 692 data points for each sample. The analysis was carried out in duplicate and all measurements were performed by the same operator.

The WinISI III (v. 1.6) software program was employed for spectral data analysis and development of chemometric models. Prior to calibration, log (1/R) spectra were corrected for the effects of scatter using the standard normal variate (SNV) and detrend (DT) algorithms and by multiplicative scatter correction (MSC) to reduce the effects of the particle size. The calibrations were performed by the modified partial least squares regression using first and second derivatives of the spectra and cross-validation was applied to optimize calibration models and to detect outliers. A total of eight spectral models for faecal nitrogen were developed, resulting from the evaluation of four scatter correction techniques (SNV; DT; SNV+DT; MSC) and two math treatments (1,4,4,1; 2,4,4,1—derivate number, subtraction gap, first smooth, second smooth). In order to obtain better accuracy in calibration, we used the mean of the two scans of each sample (average spectral data).

The performance of the model was determined by the following statistics: standard error of calibration (SEC), standard error of prediction (SEP), coefficient of determination for calibration (R^2^), coefficient of determination for validation (r^2^), the ratio of performance to deviation (RPD, defined as the ratio of standard deviation for the validation samples to the value of SEP), and the range error ratio (RER, defined as the ratio of the range in the reference data from the validation set to the SEP). The RPD ≥ 3.0 and/or RER > 10 indicate good predictions and that the equation can be used for quantitative analysis [42,43].

A total of 345 faecal samples from five herbivore species (European rabbit, horse, Pyrenean chamois, red deer, and sheep) (Table 2) were used to perform independent calibration equations for each species (monospecies calibrations) and later a multispecies calibration for samples from all species together. Internal ‘leave-n-out’ cross-validation was used as an initial test to validate all calibrations. In addition, sets of external validations were performed for each equation, for both monospecies and multispecies equations. For external validations we employed samples not included in the calibration equations. We used approximately 80% of the total samples to form the calibration sets, and around 20% for the external validation sets (Table 2). The same faecal samples were used as a validation set for the monospecies and the multispecies calibration equations. To check the predictive accuracy of the multispecies equation, samples from other species originally excluded from the multispecies equation, were employed as an independent prediction set (Table 2).

**Table 2:** Summary of nitrogen content in faecal samples used in calibration and validation sets. The results are expressed in % of dry matter.

	Calibration set				Validation set			
	N	Range	Mean	SD	n	Range	Mean	SD
**Monospecies**								
European rabbit	60	0.75–2.74	1.288	0.569	14	0.69–3.25	1.392	0.746
Horse	60	1.03–2.30	1.606	0.309	10	1.12–2.61	1.665	0.425
Pyrenean chamois	68	1.53–3.58	2.233	0.469	17	1.62–4.10	2.408	0.589
Red deer	77	1.52–3.39	2.432	0.492	19	1.61–3.31	2.375	0.502
Sheep	80	1.47–3.07	1.977	0.332	20	1.68–2.39	2.000	0.191
**Multispecies**								
E. rabbit + Horse + P. chamois + Red deer + Sheep	345	0.75–3.58	1.945	0.598	80	0.69–4.10	2.028	0.630
**Other species**	**External validation set**							
Alpine ibex	12	1.99–3.65	2.737	0.479				
Cattle	12	2.32–3.31	2.870	0.267				
European mouflon	15	1.92–4.09	3.190	0.708				
Goat	12	1.87–2.60	2.267	0.234				
Roe deer	20	1.59–3.51	2.144	0.536				

*N* = number of samples for calibration, *n* = number of samples for external validation, *Range* = interval between the maximum and minimum value of data set, *SD* = standard deviation.

### Statistical analysis

We used linear models to explore whether the relationship between NIRS predictions and laboratory FN estimates varied across species. We fitted a series of linear models in which laboratory FN values (the response variable) were explained by NIRS predictions, the herbivore species and their two-way interactions (explanatory fixed factors). Model selection was performed using the Akaike Information Criterion (AIC) corrected for small sample sizes (AICc). The AIC may perform poorly if there are too many parameters in relation to the size of the sample. The use of AICc is recommended when the ratio sample size/number of parameters is small (e.g., < 40)[44–46]. The Akaike weight (*W*i) for each competing model, i.e., the relative likelihood of the model given the data available, was also calculated. Once the best model was selected, linear model assumptions (mainly homoscedasticity and normality) were evaluated following Zuur et al. [47].

The Bland-Altman method was also used to check the agreement between monospecies and multispecies calibrations predicting FN. The Bland-Altman method uses the standard deviation and the mean of the differences between two quantitative measurements by constructing limits of agreement [48,49]. The resulting graph is a scatter plot XY, where the Y axis shows the difference between monospecies and multispecies predictions of FN, and the X axis represents the average of these predictions of FN. In addition, the Bland-Altman method proposes that 95% of the measurements should be within ±1.96 sd of the mean difference. On the other hand, the variability of the differences between both methods should be distributed within the limits of agreement and without any trend in relation to concentration of the unit of measure (i.e., percentage of FN). The Bland-Altman method was applied using the “BlandAltmanLeh” package version 0.3.1 [50]. All statistical analyses were performed using R version 3.3.2 [51].

## Results

### Analysis of NIR spectrum

**Fig 1:**
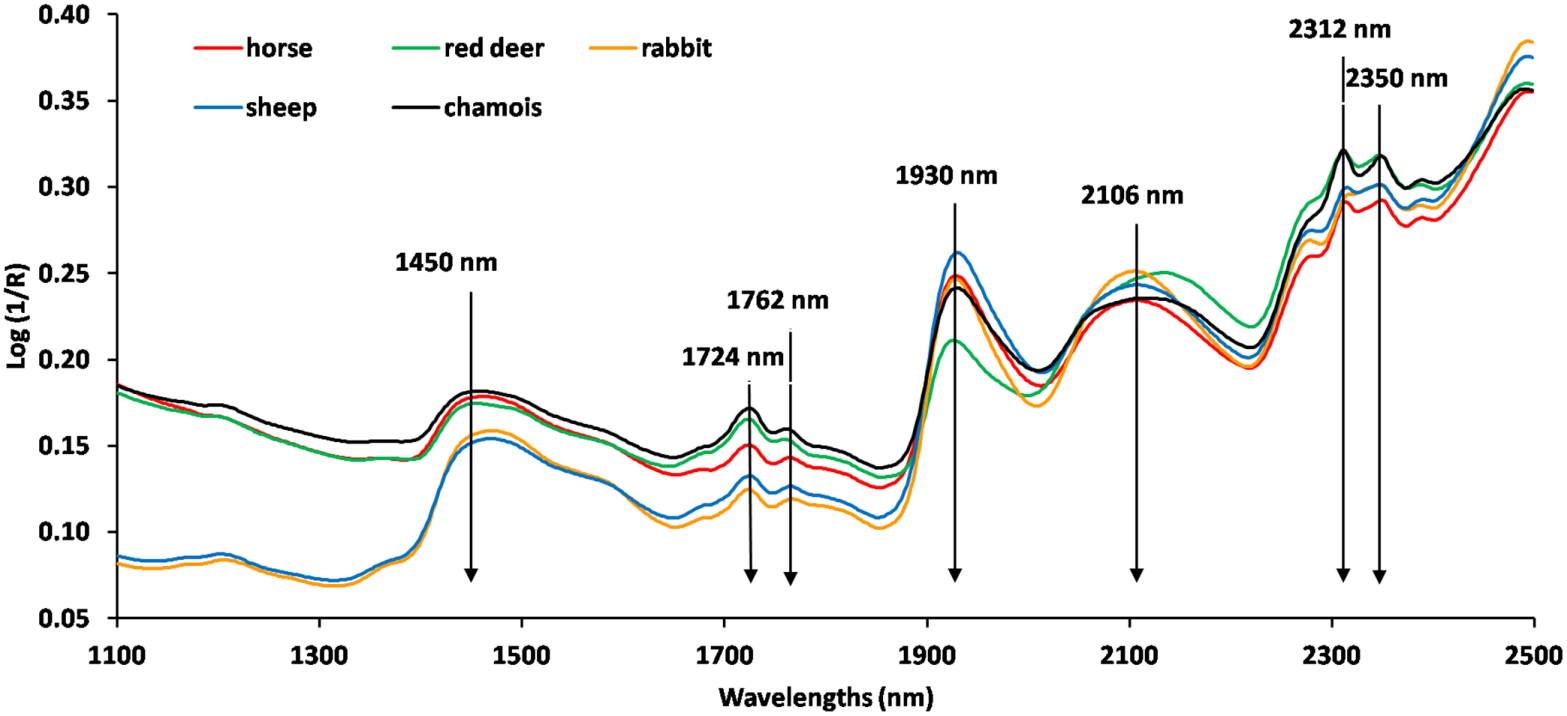
Near infrared reflectance spectra of faecal samples from five herbivorous mammalian species used to build a multispecies calibration for faecal nitrogen.

The NIR spectra of faecal samples averaged for each animal species are shown in Fig 1 (see S1 Dataset for raw data). The log (1/R) reflectance spectra of faecal samples were similar to those reported for domestic ruminants [18,52,53] and rabbits [54]. The following characteristic bands were identified: 1450 nm (1st overtone of the O-H stretch, characteristic of water content); the region 1700–1762 nm (1st overtone of C-H stretch of CH_2_ and CH_3_ groups, associated with fat content); 1930 nm (a combination of the O-H bend and the stretching band of water) and the bands 2106, 2312, and 2350 nm (combination bands of C-O stretching and bending vibrations associated with the absorption of starch and protein) [55,56]. The FN content was visible in the 2100–2350 nm region. The effect of nitrogen content was evident in reduced absorption and a shift in peaks at 2312 and 2350 nm.

#### Calibrations and validations of NIRS mono- and multispecies equations

Descriptive statistics of the faecal data set employed for the development of the calibration and validation equations are shown in Table 2. Table 3 shows the statistical results obtained from the calibration equations and the corresponding validations (both cross and external validations) carried out by NIRS. We found high and similar predictive power with both monospecies and multispecies calibrations (monospecies: R^2^ > 0.95, r_cv_^2^ > 0.93, and r^2^ > 0.88; multispecies: R^2^ = 0.98, r_cv_^2^ = 0.97, and r^2^ = 0.97). These statistics suggest that the performance of the multispecies equation was in line with the monospecies equations. The statistics RPD and RER were used as diagnostics indices [56,57]. For monospecies calibration equations, the RPD range was between 2.7 and 7.9, and the RER range was defined between 9.9 and 27.0, represented in both RPD and RER by sheep as the minimum value, and European rabbit as the maximum. In the multispecies calibration, RPD and RER were 5.3 and 28.4, respectively.

**Table 3:** Calibration and validation statistics of monospecies and multispecies calibrations used to determine the faecal nitrogen content (%) in faecal samples by NIRS analysis.

	Calibration				Cross validation		External validation					
	Math ^a^ treatment	Scatter ^b^ correction	R^2^	SEC	r_cv_^2^	SECV	r^2^	SEP	Bias	Slope	RPD	RER
**Monospecies**												
European rabbit	1,4,4,1	MSC	0.99	0.059	0.98	0.082	0.98	0.095	-0.026	0.991	7.9	27.0
Horse	2,4,4,1	MSC	0.98	0.049	0.94	0.074	0.97	0.116	0.018	1.289	3.7	12.9
Pyrenean chamois	1,4,4,1	MSC	0.98	0.080	0.96	0.101	0.97	0.100	0.006	1.038	5.9	24.8
Red deer	1,4,4,1	SNV+D	0.95	0.107	0.94	0.121	0.92	0.152	-0.019	1.106	3.3	11.2
Sheep	1,4,4,1	MSC	0.96	0.061	0.93	0.074	0.88	0.072	0.028	0.988	2.7	9.9
**Multispecies**												
E. rabbit + Horse + P. chamois + Red deer + Sheep	1,4,4,1	SNV+D	0.98	0.087	0.97	0.098	0.97	0.120	0.033	0.933	5.3	28.4

^a^ *Math treatment*: derivative order, subtraction gap, first smoothing, second smoothing;

^b^ *SNV* = standard normal variate, D = detrend, *MSC* = multiple scatter correction. *R*^*2*^ = coefficient of determination for calibration, *SEC* = standard error of calibration, *r*_*cv*_^*2*^ = coefficient of determination for cross validation, *SECV* = standard error of cross validation, *r*^*2*^ = coefficient of determination for external validation, *SEP* = standard error of prediction, *RPD* = ratio of performance to deviation (SD/SEP) and *RER* = range error ratio (Range/SEP).

To test the reliability of the multispecies equation an external validation was performed with faecal samples not involved in the multispecies calibration. The aim was to check the FN prediction of samples from each species individually using the multispecies equation (Table 4). The results of the predictions showed an r^2^ between 0.77 and 0.97, with red deer having the lowest value and European rabbit and Pyrenean chamois the highest. The predictive power of the equation was consistently better for samples from European rabbit, horse and Pyrenean chamois than for sheep and red deer. Comparing predictions for monospecies (Table 3) and for each species using the multispecies equation (Table 4), the lowest RPD and RER values were represented by red deer and sheep, whereas the highest RPD and RER were in European rabbit.

**Table 4:** Validation statistics using the multispecies equation to predict the faecal nitrogen content (% of dry matter) in faecal samples of each species.

		External validation					
	N	r^2^	SEP	Bias	Slope	RPD	RER
European rabbit	14	0.97	0.130	0.031	0.948	5.7	19.7
Horse	10	0.95	0.124	0.072	1.129	3.4	12.0
Pyrenean chamois	17	0.97	0.113	0.023	1.061	5.2	22.0
Red deer	19	0.77	0.245	-0.049	0.944	2.1	6.9
Sheep	20	0.89	0.078	0.039	0.877	2.5	9.1

*r*^*2*^ = coefficient of determination for external validation, *SEP* = standard error of prediction, *RPD* = ratio of performance to deviation (SD/SEP), *RER* = range error ratio (Range/SEP).

We used the multispecies equation to assess whether it was possible to predict the FN of new species of herbivores and to therefore check the multispecies equation. To this end, we tested faecal samples of Alpine ibex, cattle, European mouflon, domestic goat and roe deer for prediction (Table 5). The results obtained were relatively good. The r^2^ values were greater than 0.86 and SEP ranged between 0.08 and 0.27, with the boundaries given by goat and European mouflon, respectively. The RPD ranged between 2.6 and 3.2, whereas the RER ranged between 8.1 and 11.1, with the Alpine ibex the best predicted, and the European mouflon the poorest.

**Table 5:** NIRS predicted faecal nitrogen contents (% of dry matter) in faecal samples and validation statistics using the multispecies equation.

Validation species	NIRS predicted values			Statistics for NIRS predictions					
	Range	Mean	SD	r^2^	SEP	Bias	Slope	RPD	RER
Alpine ibex	2.09–3.39	2.687	0.411	0.93	0.150	0.037	1.173	3.2	11.1
Cattle	2.30–3.37	2.881	0.281	0.86	0.101	-0.009	0.902	2.6	9.8
European mouflon	2.02–3.94	3.242	0.615	0.86	0.267	-0.053	1.066	2.7	8.1
Goat	1.71–2.48	2.214	0.249	0.92	0.087	0.053	0.901	2.7	8.4
Roe deer	1.70–3.45	2.295	0.528	0.91	0.204	-0.132	0.985	2.6	9.4

Range = interval between the maximum and minimum value of the data set, SD = standard deviation, r^2^ = coefficient of determination for prediction, SEP = standard error of prediction, RPD = ratio of performance to deviation (= SD/SEP) and RER = range error ratio (= Range/SEP).

### Relationship between NIRS predictions and laboratory FN estimates among species

**Fig 2:**
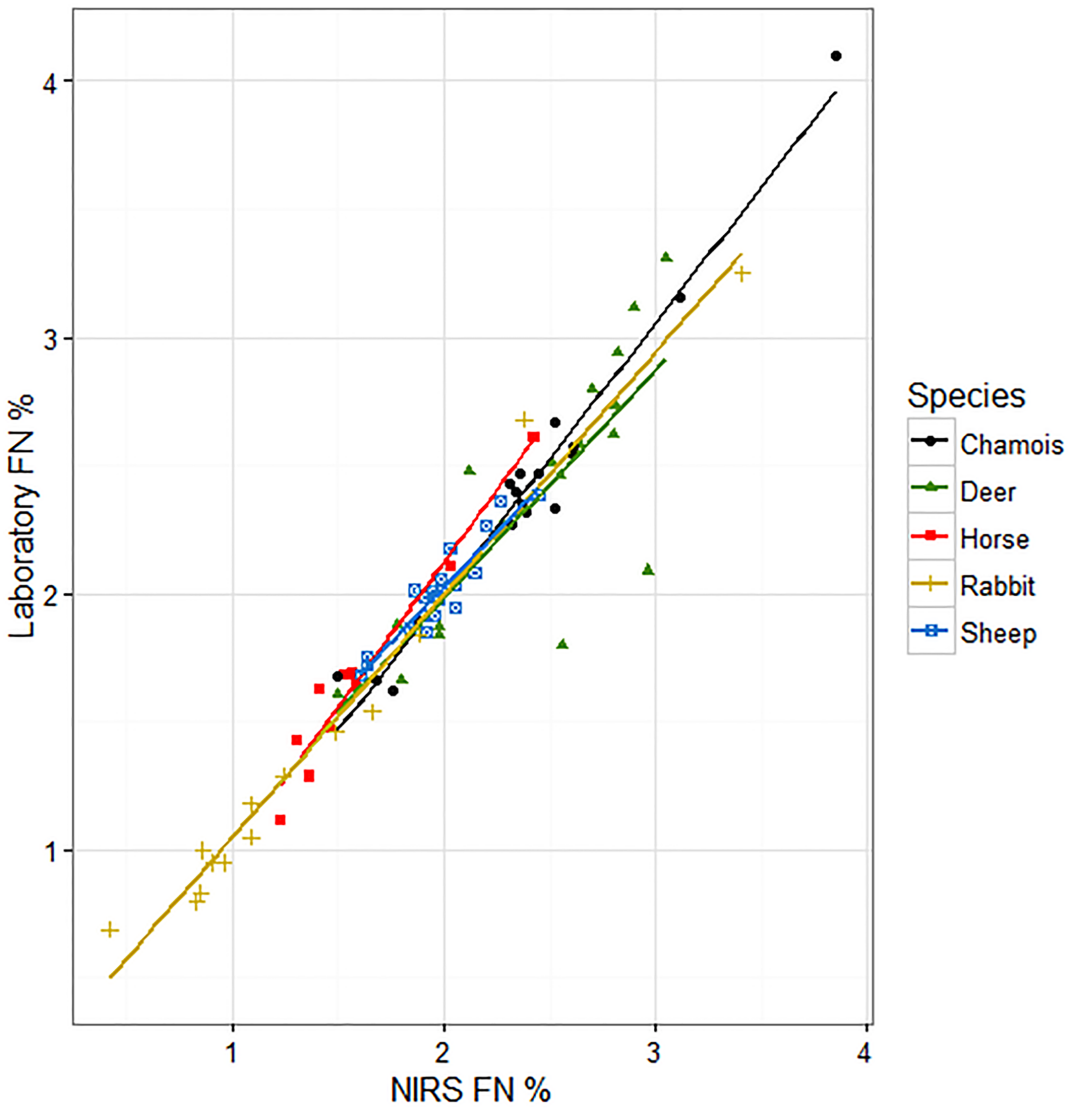
Relationships between faecal nitrogen (FN) predicted by NIRS and FN estimated by the Dumas dry combustion method in five herbivorous mammal species.

The best model to explain the observed variability of laboratory FN included the effects of NIRS predictions and the herbivore species (R^2^ = 0.92, *W*i = 0.89; Table 6). It indicates that the slope between laboratory FN and NIRS calibrations was similar for all species varying only in the intercept (Fig 2, see S1 Dataset for raw data). Thus, we can assume a single relationship between NIRS FN predictions and laboratory FN in all species considered.

**Table 6:** Model selection to explore whether the relationships between faecal nitrogen (FN) predicted by NIRS and faecal nitrogen estimated by the Dumas dry combustion method (reference method) varied among species.

Biological Models	K	AICc	Δi	*W*i
**FN laboratory + Species**	7	-755.43	0.00	0.89
FN laboratory * Species	11	-750.92	4.51	0.09
FN laboratory	3	-747.26	8.17	0.02

K = number of parameters, AICc = Akaike Information Criterion corrected for small sample sizes, Δi = difference of AICc with respect to the best model, *W*i = Akaike weight. The best model is indicated in bold.

**Fig 3:**
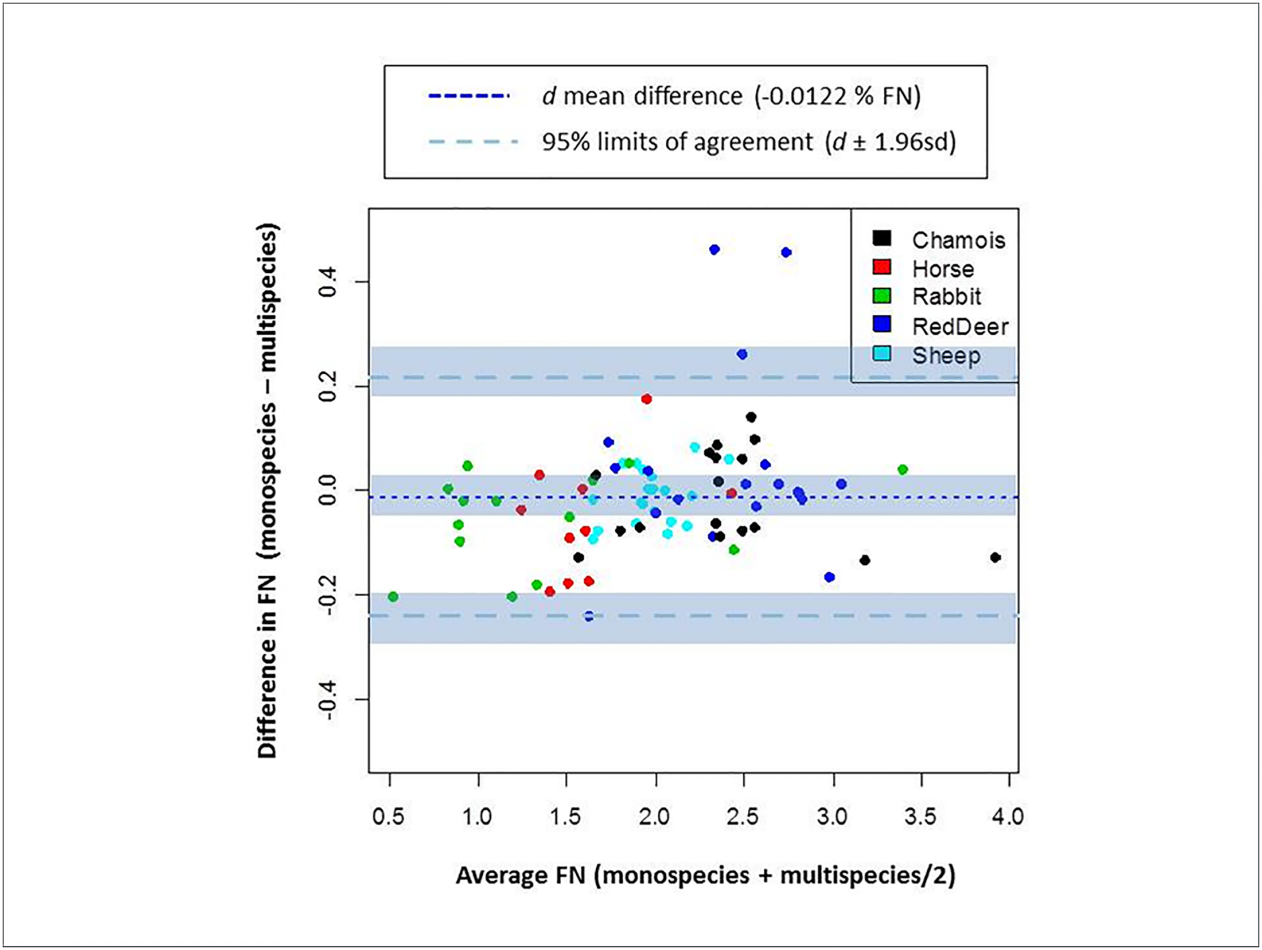
Bland and Altman plot of the difference between mono- and multispecies NIRS calibrations to predict the faecal nitrogen (FN expressed on a % dry matter basis) in five herbivorous mammal species. The mean of differences (*d*) between mono- and multispecies calibration equations are represented by a dotted dark-blue line, whereas limits of agreement (*d* ± 1.96sd) are represented by dashed lines. Confidence intervals at 95% are shown by shaded areas.

Moreover, our Bland-Altman analysis showed that the mean of differences (*d*) between monospecies and multispecies predictions (i.e., bias) was -0.0122, and the limits of agreement were *d* + 1.96sd = 0.2167 and *d* − 1.96sd = -0.2411 (Fig 3). Also, the confidence intervals (95%) were calculated for both the mean of differences and the limits of agreement. The variability of the differences of FN between the two calibrations was distributed within the limits of agreement and without any tendency in relation to the mean of FN (i.e., mean of monospecies and multispecies predictions for FN). From 80 samples in total, just 3 animals (all red deer) were outside the limits of agreement.

## Discussion

We have demonstrated that a single multispecies NIRS calibration can be used to assess the FN content in faeces of a broad range of herbivore species. In fact, the accuracy of calibration and validation procedures, as well as the prediction values derived from the multispecies equation were similar to those observed in monospecies equations. Comparing multispecies and monospecies calibrations, the coefficients of determination and standard error values were very similar for European rabbit, horse, and Pyrenean chamois and even better for red deer and sheep. Acceptable faecal NIRS equations must have R^2^ > 0.8, SECV close to SEC, and RPD > 3 [43,56], criterion which were achieved by our multispecies calibration.

In accordance with the criteria of Shenk and Westerhaus [57], our multispecies NIRS calibration for FN was excellent, and had greater predictive ability than previously reported monospecies calibrations, which were more accurate than the monospecies calibrations reported in the literature for faeces of herbivores [18]. The SECV values for FN were very low indicating that the prediction obtained by the NIRS multispecies model was highly accurate. This error value of cross validation was even lower than those found in other equations built for faecal crude protein in wild ungulates, such as red deer and roe deer (0.15 and 0.12, respectively; [58]), in free-ranging goats (0.13; [33]), or livestock animals such as sheep (0.15; [59]) and domestic rabbit using protein rich compound feeds (0.22; [54]).

Additionally, we also considered other statistics, such as SEP, to measure the calibration performance and to estimate the fit between predicted and reference values when the multispecies equation is applied to an external validation data set (samples from species not used in the calibration). This indicator of prediction error, and related indicators such as RPD and RER, provide information about the reliability of the calibration and validation [42,43,60,61]. On the one hand, in all species, except red deer, we found very similar values of SEP between monospecies predictions and multispecies predictions using the corresponding external validation set. In relation to the literature, the SEP values from the multispecies prediction for each individual species were similar or even greater than those reported for dietary nitrogen based on deer (SEP = 0.15; [62]) or sheep (SEP = 0.33; [59]). On the other hand, multispecies validation showed RPD and RER values generally higher than the minimum considered suitable for faecal samples (i.e., RPD > 3 and RER > 10). The individual predictions using the multispecies equation show diverse RER and RPD depending on the species. In the case of sheep (RPD = 2.5; RER = 9.1) and red deer (RPD = 2.1; RER = 6.9), the statistics were close to the minimum requirements, possibly because of the low value of the standard deviation for sheep and the high value of SEP for red deer compared to other species. Along the same lines, the Bland Altman analysis confirmed that both mono- and multispecies calibrations can be interchangeable to predict FN in the species studied.

We have shown that it is possible to use a single NIRS calibration equation to predict the FN of several herbivorous species (horse, European rabbit, Pyrenean chamois and sheep), thus avoiding the need to perform species specific calibrations. Some studies [40,54,63] have employed multispecies calibration but mainly with forages or different feed compounds. An example of using a multispecies calibration equation in faeces was demonstrated by Decruyenaere et al. [63], where databases with a diversity of plant species were used to predict digestibility in sheep. Nuñez et al. [54] also used different diets as a calibration set to predict digestibility parameters, as well as the crude protein in faeces, of domestic rabbits. However, to the best of our knowledge, no previous study has developed a multispecies equation to predict the FN of animal species.

The fact that the slope of the relationship between NIRS FN predictions and laboratory FN was similar for each species, supports the possibility of using a single global equation to predict the faecal nitrogen in a broad range of herbivores. We have shown that a calibration built with samples from a wide diversity of herbivores (large range of FN) can predict with accuracy the FN values in samples from other herbivores when the FN values are within the FN range of the data set used in the multispecies calibration. Additionally, we could have used a single multispecies equation instead of five monospecies equations for each of the herbivore species to predict the FN by NIRS.

The most likely explanation for the success of the multispecies calibration is based on the “amplitude spectral” concept. Studies of NIRS-based predictions of forage quality using monospecies calibrations are often, but not always, more accurate than multispecies calibrations that are aimed to encompass a wider spectral variety and range. Marten et al. [36] concluded that several species of legumes can be evaluated simultaneously with a single calibration equation nearly as well as can the single species. There may be a trade-off between the robustness of a calibration in terms of its ability to predict attributes in sets of samples that exhibit wide variability, and the accuracy of those predictions is reflected in the SEP values. Consequently, a compromise exists between having a calibration set with a large number of samples and wide spectral variability, and a lower quality of prediction reflected by higher error as seen in the monospecies calibrations. If the aim is to predict samples with high spectral variability, such as in the case of seasonal variation in FN, it is much better to use a multispecies calibration equation. Conversely, if the predicted samples are always from the same animal species and have similar spectral variability, it is better to use a monospecies calibration equation.

To evaluate the quality of the multispecies calibration, we tested the equation using samples from species not included in the equation, namely Alpine ibex, cattle, domestic goat, European mouflon, and roe deer. The multispecies calibration equation could be applied to predict the FN in other species of herbivores, thus avoiding the need to produce new and specific monospecies calibrations. This approach is quite useful when it is necessary to analyse only a few samples and when the minimum number of samples necessary to develop an individual NIRS calibration equation with accuracy is not achievable.

In summary, we can predict the FN of a wide variety of herbivores using a single calibration equation if a broad spectral variety of the data set is used for the calibration equation. Indeed, the prediction of FN in herbivore species that are not included in the calibration is possible, whether or not the new species present a range of FN within the FN range used in the calibration equation.

## Supporting information

S1 Dataset Raw data containing laboratory, NIRS-predicted faecal nitrogen values and NIRS spectra values by species. (XLS)

## References

[1] Kohn M, Wayne R. Facts from feces revisited. Trends Ecol Evol. 1997;12: 223–227. 21238046 10.1016/s0169-5347(97)01050-1

[2] Putman RJ. Facts from faeces. Mamm Rev. 1984;14: 79–97.

[3] Poinar H, Hofreiter M, Spaulding W, Martin P, Stankiewicz B, Bland H, et al Molecular coproscopy: dung and diet of the extinct ground sloth nothrotheriops shastensis. Science (80-). 1998;281: 402–406. 10.1126/science.281.5375.402 9665881

[4] Rivera L, Baraza E, Alcover JA, Bover P, Rovira CM, Bartolome J. Stomatal density and stomatal index of fossil Buxus from coprolites of extinct *Myotragus balearicus* Bate (Artiodactyla, Caprinae) as evidence of increased CO_2_ concentration during the late Holocene. The Holocene. 2014;24: 876–880.

[5] McCaig TN. Extending the use of visible/near-infrared reflectance spectrophotometers to measure colour of food and agricultural products. Food Res Int. 2002;35: 731–736.

[6] Hibert F, Maillard D, Fritz H, Garel M, Abdou HN, Winterton P. Ageing of ungulate pellets in semi-arid landscapes: How the shade of colour can refine pellet-group counts. Eur J Wildl Res. 2011;57: 495–503.

[7] Bubb A, Ehlers K, Kotze A, Grobler PP. The effect of sample age and storage method on DNA yield and microsatellite amplification from baboon (*Papio ursinus*) faecal samples. Eur J Wildl Res. 2011;57: 971–975.

[8] Kamler J, Homolka M, Kráčmar S. Nitrogen characteristics of ungulates faeces: Effect of time of exposure and storage. Folia Zool. 2003;52: 31–35.

[9] Jean PO, Bradley RL, Tremblay JP, Cote SD. Combining near infrared spectra of feces and geostatistics to generate forage nutritional quality maps across landscapes. Ecol Appl. 2015;25: 1630–1639. 26552270 10.1890/14-1347.1

[10] Barnier F, Valeix M, Duncan P, Chamaillé-Jammes S, Barre P, Loveridge AJ, et al Diet quality in a wild grazer declines under the threat of an ambush predator. Proc Biol Sci. 2014;281: 20140446 10.1098/rspb.2014.0446 24789903 PMC4024301

[11] Villamuelas M, Fernández N, Albanell E, Gálvez-Cerón A, Bartolomé J, Mentaberre G, et al The Enhanced Vegetation Index (EVI) as a proxy for diet quality and composition in a mountain ungulate. Ecol Indic. Elsevier Ltd; 2016;61: 658–666.

[12] Foley WJ, McIlwee A, Lawler I, Aragones L, Woolnough AP, Berding N. Ecological applications of near infrared reflectance spectroscopy—a tool for rapid, cost-effective prediction of the composition of plant and animal tissues and aspects of animal performance. Oecologia. 1998;116: 293–305. 10.1007/s004420050591 28308060

[13] Stuth J, Jama A, Tolleson D. Direct and indirect means of predicting forage quality through near infrared reflectance spectroscopy. F Crop Res. 2003;84: 45–56.

[14] Vance CK, Tolleson DR, Kinoshita K, Rodriguez J, Foley WJ. Near infrared spectroscopy in wildlife and biodiversity. J Near Infrared Spectrosc. 2016;25: 1–25.

[15] Hewison AJM, Morellet N, Verheyden H, Daufresne T, Angibault JM, Cargnelutti B, et al Landscape fragmentation influences winter body mass of roe deer. Ecography (Cop). 2009;32: 1062–1070.

[16] Holecheck J, Vavra M, Pieper R. Methods for determining the nutritive quality of range ruminant diets: a review. J Anim Sci. 1982;54: 363–376.

[17] Robbins CT. Wildlife feeding and nutrition. London: Academic Press; 1983.

[18] Dixon R, Coates D. Review: Near infrared spectroscopy of faeces to evaluate the nutrition and physiology of herbivores. J Near Infrared Spectrosc. 2009;17: 1–31.

[19] Lyons RK, Stuth JW, Angerer JP. Technical Note : Fecal NIRS equation field validation. 1995;48: 380–382.

[20] Orskov ER. Protein nutrition in ruminants. London: Academic Press; 1982.

[21] Clauss M, Steuer P, Müller DWH, Codron D, Hummel J. Herbivory and body size: Allometries of diet quality and gastrointestinal physiology, and implications for herbivore ecology and dinosaur gigantism. PLoS One. 2013;8. 10.1371/journal.pone.0068714 PMC3812987 24204552

[22] Leslie DMJ, Bowyer RT, Jenks JA. Facts from feces: Nitrogen still measures up as a nutritional index for mammalian herbivores. J Wildl Manage. 2008;72: 1420–1433.

[23] Gálvez-Cerón A, Serrano E, Bartolomé J, Mentaberre G, Fernández-Aguilar X, Fernández-Sirera L, et al Predicting seasonal and spatial variations in diet quality of Pyrenean chamois (*Rupicapra pyrenaica pyrenaica*) using near infrared reflectance spectroscopy. Eur J Wildl Res. 2013;59: 115–121.

[24] Gil-Jiménez E, Villamuelas M, Serrano E, Delibes M, Fernández N. Fecal nitrogen concentration as a nutritional quality indicator for European rabbit ecological studies. PLoS One. 2015;10: 1–14. 10.1371/journal.pone.0125190 PMC4404320 25893872

[25] Mésochina P, Martin-Rosset W, Peyraud J, Duncan P, Micol D, Boulot S. Prediction of the digestibility of the diet of horses: evaluation of faecal indices. Grass Forage Sci. 1998;53: 189–196.

[26] Wang CJ, Tas BM, Glindemann T, Rave G, Schmidt L, Weißbach F, et al Fecal crude protein content as an estimate for the digestibility of forage in grazing sheep. Anim Feed Sci Technol. 2009;149: 199–208.

[27] Blanchard P, Festa-Bianchet M, Gaillard JM, Jorgenson JT. A test of long-term fecal nitrogen monitoring to evaluate nutritional status in bighorn sheep. J Wildl Manage. 2003; 477–484.

[28] Gálvez-Cerón A, Gassó D, López-Olvera JR, Mentaberre G, Bartolomé J, Ignasi M, et al Gastrointestinal nematodes and dietary fibre: Two factors to consider when using FN for wildlife nutrition monitoring. Ecol Indic. 2015;52: 161–169.

[29] Barboza PS, Parker KL, Hume ID. Integrative Wildlife Nutrition. Berlin, Heidelberg (Germany): Springer-Verlag; 2009.

[30] Hanley TA. The Nutritional Basis for Food Selection by ungulates. J Range Manag. 1982;35: 146–151.

[31] Huston J, Rector B, Ellis W, Allen M. Dynamics of Digestion in Cattle, Sheep, Goats and Deer. J Anim Sci. 1986;62: 208–215. 3957805 10.2527/jas1986.621208x

[32] Van Soest PJ. Nutritional ecology of the ruminant. Cornell University Press; 1994.

[33] Leite ER, Stuth JW. Fecal NIRS equations to assess diet quality of free-ranging goats. Small Rumin Res. 1995;15: 223–230.

[34] Marten GC, Shenk JS, Barton FE. Near infrared reflectance spectroscopy (NIRS): Analysis of forage quality. USA: Agriculture handbook; 1989.

[35] Shenk JS, Westerhaus MO, Hoover MR. Analysis of Forages by Infrared Reflectance. J Dairy Sci. 1979;62: 807–812.

[36] Marten GC, Brink GE, Buxton DR, Halgerson JL, Hornstein JS. Near infrared reflectance spectroscopy analysis of forage quality in four legume species. Crop Sci. 1984;24: 1179–1182.

[37] Locher F, Heuwinkel H, Gutser R, Schmidhalter U. Development of near infrared reflectance spectroscopy calibrations to estimate legume content of multispecies legume-grass mixtures. Agron J. 2005;97: 11–17.

[38] Norman HC, Hulm E, Humphries AW, Hughes SJ, Law R, Rowe T, et al. Broad NIRS calibrations to predict nutritional value of the southern feedbase. 17th ASA Conference Building Productive, Diverse and Sustainable Landscapes. Hobart, Australia; 2015. pp. 3–6.

[39] Belanche A, Weisbjerg MR, Allison GG, Newbold CJ, Moorby JM. Estimation of feed crude protein concentration and rumen degradability by fourier-transform infrared spectroscopy. J Dairy Sci. 2013;96: 7867–7880. 10.3168/jds.2013-7127 24094538

[40] Foskolos A, Calsamiglia S, Chrenková M, Weisbjerg MR, Albanell E. Prediction of rumen degradability parameters of a wide range of forages and non-forages by NIRS. Animal. 2015;9: 1163–1171. 10.1017/S1751731115000191 25692809

[41] AOAC. Official methods of analysis. 19th ed Latimer G. W., editor. Gaithersburg (MD): Association of Official Analytical Chemists International; 2012.

[42] Williams PC, Sobering DC. How do we do it: a brief summary of the methods we use in developing near infrared calibrations Near infrared spectroscopy: The future waves. 1996 pp. 185–188.

[43] Williams P. Tutorial: The RPD statistic: a tutorial note. NIR news. 2014;25: 22.

[44] Burnham KP, Anderson DR. Model selection and multimodel inference: a practical information-theoretic approach. 2nd ed New York: Springer-Verlag; 2002.

[45] Johnson JB, Omland KS. Model selection in ecology and evolution. Trends Ecol Evol. 2004;19: 101–108. 10.1016/j.tree.2003.10.013 16701236

[46] Akaike H. A New Look at the Statistical Model Identification. IEEE Trans Automat Contr. 1974;19: 716–723.

[47] Zuur AF, Ieno EN, Smith GM. Analysing Ecological Data. Springer Science & Business Media, editor. 2007.

[48] Altman DG, Bland JM. Measurement in medicine: The analysis of method comparison studies. Statistician. 1983;32: 307–317.

[49] Giavarina D. Lessons in Biostatistics. Biochem Medica. 2015;25: 141–151. 10.11613/BM.2015.015 PMC4470095 26110027

[50] Lehnert B. BlandAltmanLeh: plots (slightly extended) Bland-Altman plots. R Packag version 031 https://CRANR-project.org/package=BlandAltmanLeh. 2015;

[51] Team R. A language and environment for statistical computing. Vienna, Austria: R Foundation for Statistical Computing; 2014.

[52] Coleman SW, Murray I. The use of near-infrared reflectance spectroscopy to define nutrient digestion of hay by cattle. Anim Feed Sci Technol. 1993;44: 237–249.

[53] Windham W, Hill N, Stuedemann J. Ash in forage, esophageal, and fecal samples analyzed using near-infrared reflectance spectroscopy. Crop Sci. 1991;31: 1345–1349.

[54] Núñez-Sánchez N, Martínez Marín AL, Hernández MP, Carrion D, Castro GG, Pérez Alba LM. Faecal near infrared spectroscopy (NIRS) as a tool to asses rabbit’s feed digestibility. Livest Sci. 2012;150: 386–390.

[55] Murray I, Williams PC. Chemical Principles of Near-Infrared Technology In: Williiams PC, Norris KH, editors. Near-Infrared Technology in the Agricultural and Food Industries. St.Paul (MN): AACC Inc.; 1987 pp. 17–34.

[56] Williams PC. Implementation of near-infrared technology In: Williams PC, Norris KH, editors. Near-iIfrared Technology in the Agricultural and Food Industries. 2nd Editio St Paul (MN); 2001 pp. 143–167.

[57] Shenk JS, Westerhaus MO. Calibration the ISI way In: Davies AMC, Williams PC, editors. Near infrared spectroscopy: The future waves. Chichester (UK): NIR Publications; 1996 pp. 198–202.

[58] Kamler J, Homolka M. Faecal nitrogen: A potential indicator of red and roe deer diet quality in forest habitats. Folia Zool. 2005;54: 89–98.

[59] Li H, Tolleson D, Stuth J, Bai K, Mo F, Kronberg S. Faecal near infrared reflectance spectroscopy to predict diet quality for sheep. Small Rumin Res. 2007;68: 263–268.

[60] Fearn T. Assessing calibrations: SEP, RPD, RER and R^2^. NIR News. 2002.

[61] Williams P, Sobering D. Comparison of commercial near infrared transmittance and reflectance instruments for analysis of whole grains and seeds. J Near Infrared Spectrosc. 1993;1: 25–32.

[62] Showers S, Tolleson D, Stuth J, Kroll J, Koerth B. Predicting diet quality of white-tailed deer via NIRS fecal profiling. Rangel Ecol Manag. 2006;59: 300–307.

[63] Decruyenaere V, Lecomte P, Demarquilly C, Aufrere J, Dardenne P, Stilmant D, et al Evaluation of green forage intake and digestibility in ruminants using near infrared reflectance spectroscopy (NIRS): Developing a global calibration. Anim Feed Sci Technol. 2009;148: 138–156.

